# Supplementary figures and images for: Adaptation of Bacillus thuringiensis to Plant Colonization Affects Differentiation and Toxicity
Source: mSystems. 2021 Oct 12;6(5):e00864-21. doi: 10.1128/mSystems.00864-21 (PMC8510532; doi:10.1128/mSystems.00864-21)

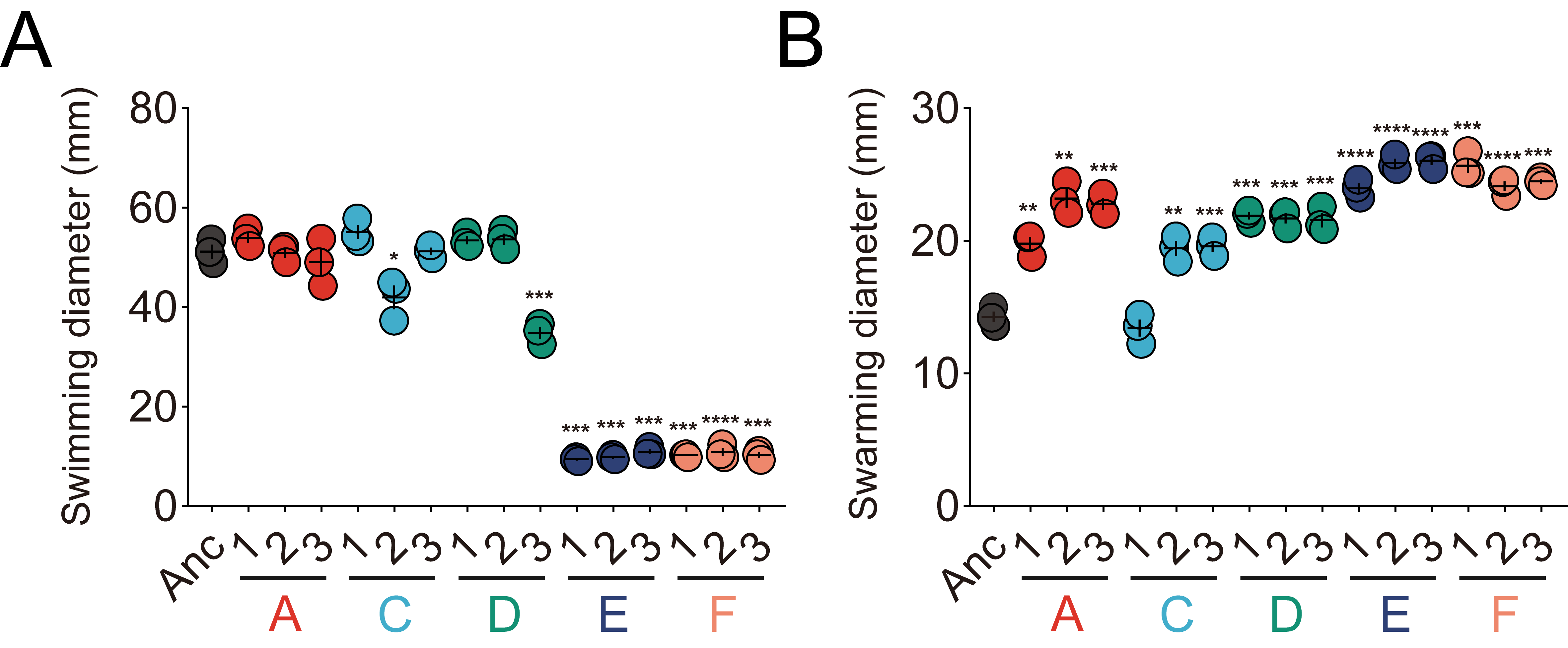

Supplement: FIG S3 [file msystems.00864-21-sf003.tif]

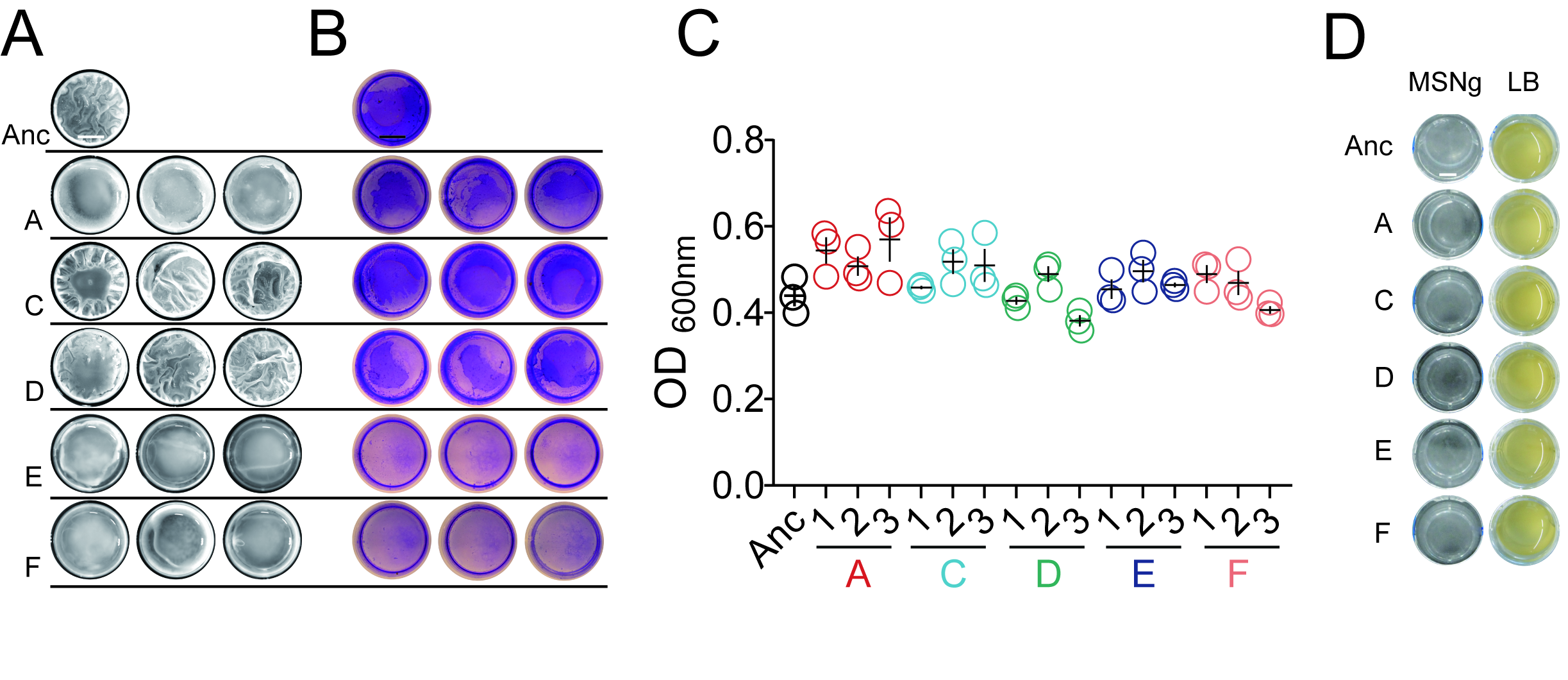

Supplement: FIG S4 [file msystems.00864-21-sf004.tif]

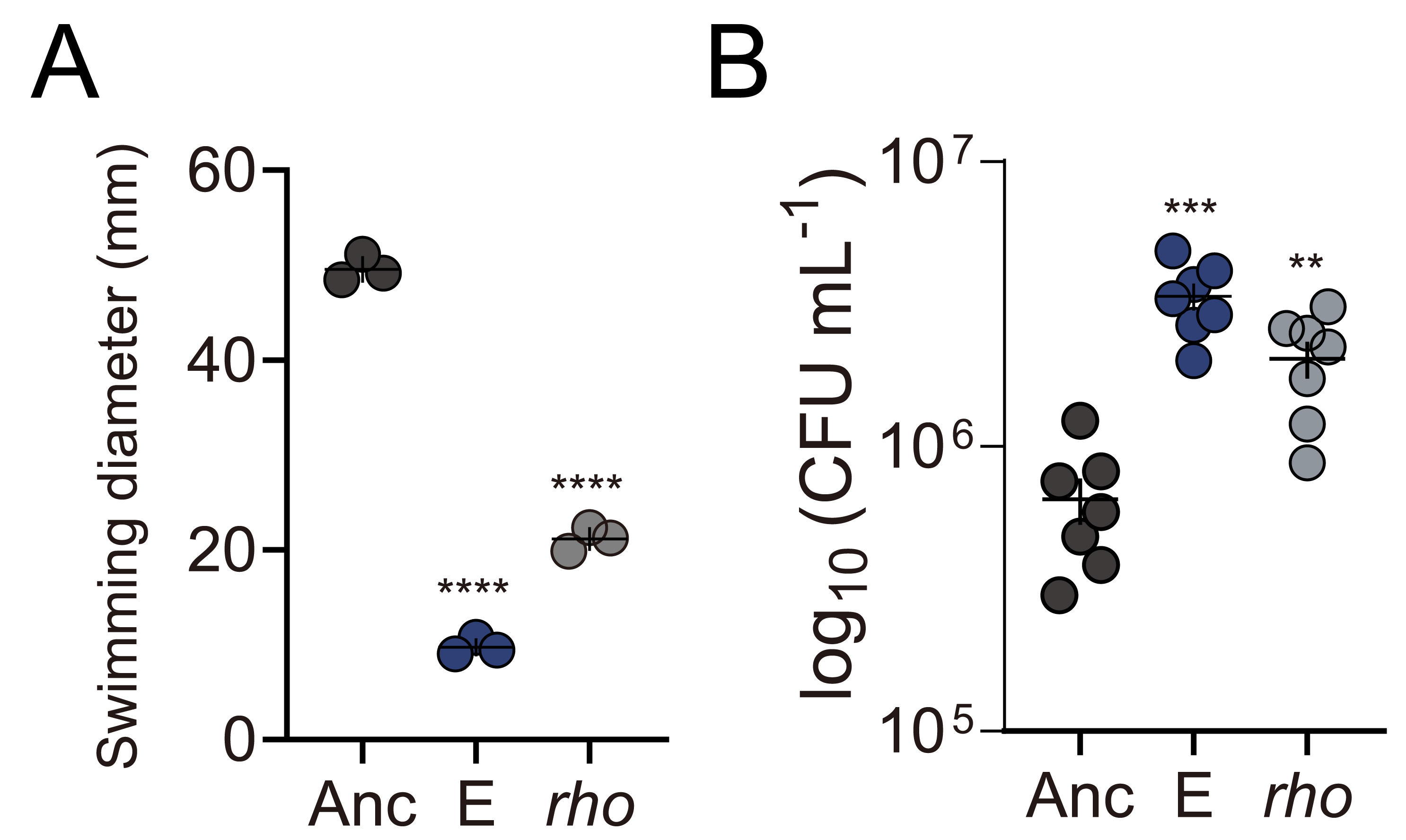

Supplement: FIG S6 [file msystems.00864-21-sf006.tif]
